# Supplementary material for: Motorization and its consequences: A mixed-methods study on the epidemiology, impact, and stakeholder perspectives of road traffic injuries among undergraduate two-wheeler riding students in Nepal
Source: PLoS One. 2026 May 27;21(5):e0341275. doi: 10.1371/journal.pone.0341275 (PMC13215494; doi:10.1371/journal.pone.0341275)
Supplement: Attachment — Submitted filename: PLOS 1 review.doc [file pone.0341275.s002.doc]

**REVIEW**

**Motorization and Its Consequences: A Mixed-Methods Study on the Epidemiology, Impact, and Stakeholder Perspectives of Road Traffic Injuries Among Undergraduate Students in Nepal**

**Over all:** This study is relevant as it addresses the rising burden of road traffic injuries among youth in Nepal, a vulnerable yet often overlooked population in public health policy. With the suggested changes, it can be considered for publication.

**Abstract**

Line 29: Methodology: to check this statement ’Methodology: The quantitative section was conducted among undergraduate students in the four colleges in Kathmandu district.’ Quantitative section?

**Introduction:**

Line 52 and 54: ‘Injuries are disproportionately high

among males, disadvantaged groups,’ add why they are disadvantaged groups.

Line 77, 78: It is mentioned 28 classes, Specify what are classes. ‘ Participation was voluntary and gave informed consent ‘ check the grammar of this statement

**Methodology:** Add timeline/ duration of the study

**Results:** Line 168, Check the grammar. Table 23 appears before table 4. Where are table 2 and 3?

**Discussion:**

Line 198 and 199: ‘no statistically significant association

was found between licensing status and RTI occurrence’. This is not reflected in results.

‘**T**he themes and recommendations from the qualitative study’ should be better discussed in this section.

The discussion could be strengthened further through more robust comparisons with national and international studies, which would help contextualize the findings, highlight unique challenges in the Nepali context, and support evidence-based recommendations.
