# Supplementary material for: Motorization and its consequences: A mixed-methods study on the epidemiology, impact, and stakeholder perspectives of road traffic injuries among undergraduate two-wheeler riding students in Nepal
Source: PLoS One. 2026 May 27;21(5):e0341275. doi: 10.1371/journal.pone.0341275 (PMC13215494; doi:10.1371/journal.pone.0341275)
Supplement: Attachment — Submitted filename: rebuttal letter with comments addressed.docx [file pone.0341275.s004.docx]

Dear Academic Editor and Reviewers,

We sincerely thank you for your thoughtful and constructive feedback on our manuscript. “Motorization and Its Consequences: A Mixed-Methods Study on the Epidemiology, Impact, and Stakeholder Perspectives of Road Traffic Injuries Among Undergraduate Two-Wheeler Riding Students in Nepal” We have carefully considered each of your comments and revised the manuscript accordingly. Below, we provide a point-by-point response to the editorial requirements and reviewer comments. All changes have been incorporated into the revised manuscript, and we believe these revisions have significantly improved the clarity and quality of our work.

We are grateful for the opportunity to revise our manuscript and for the valuable feedback provided. We hope the revisions meet your expectations and look forward to your favorable consideration.

Sincerely,

Dr. Nikita Bhattarai

(on behalf of all authors)

Please find all the point-by-point response to the editorial requirements below:

**Editorial Requirements**

**1. PLOS ONE Style Requirements and File Naming**

- We have revised the manuscript to align with PLOS ONE’s formatting guidelines, including file naming conventions, as outlined in the provided templates.

**2. Funding Information Consistency**

- We have corrected the discrepancy between the ‘Funding Information’ and ‘Financial Disclosure’ sections. The revised Funding Statement now reads:

This study was funded by the Nepal Health Research Council through the Post Graduate Health Research Grant, 2020 (Grant Number: 1585), awarded to the corresponding author, Nikita Bhattarai (NB), for her master’s thesis. The funder had no role in study design, data collection and analysis, decision to publish, or preparation of the manuscript.

- We have removed all funding-related text from the Acknowledgments section as mentioned

**3. Acknowledgments Section**

We have revised the Acknowledgments section to remove funding-related content. The updated section now reads:

- “We would like to thank all the undergraduate students who participated in our study, as well as Dr. Nishchal Dhakal, Mr. Ujjwal Upadhyay, and Father Augustine Thomas for their support in facilitating data collection at their respective colleges. We also extend our gratitude to the five key informants who generously shared their time and insights, which were invaluable to this research.”

**4. Data Availability Statement**

- We have revised the Data Availability Statement to reflect our data sharing plan. The updated statement in the cover letter :
- “All the authors have agreed and confirmed to make the underlying data available upon acceptance of the manuscript.”

**5. Author Affiliations**

- We have updated the manuscript to ensure that each author is linked to their respective institutional affiliation. Where applicable, current affiliations have been noted.

**6. Table References**

- We have corrected the numbering and referencing of all tables. Tables 1 and 2 are now appropriately cited in the main text.

**10. Reference List Review**

We have reviewed the reference list for completeness and accuracy. No retracted articles were cited. All references have been updated and formatted according to PLOS ONE guidelines.

Please find all the point by point response to the reviewer’s comments below:

**Reviewer #1 Comments**

Reviewer #1: Title – acceptable but title says about all students but result is only for two-wheeler drivers? change the title or add the data of pillion riders also if you have the data.

- The title has been changed to Motorization and Its Consequences: A Mixed-Methods Study on the Epidemiology, Impact, and Stakeholder Perspectives of Road Traffic Injuries Among Undergraduate Two-Wheeler riding Students in Nepal. To make it more clear as only the undergraduate students who were using a two wheeler were considered to be a part of the study.

Methods – is there secondary records of the police system and triangulate with your prevalence of RTI? As the prevalence you claim is lower 23%

- This study was done during the pandemic hence the prevalence of the study seems to be lower than the secondary records of the police system

Results – a response rate of 64.8% is very low and your results may be biased – justify
, 6.3% were under the influence of alcohol -is this self-reported? or was it confirmed by some agency?

- The response rate was relatively low due to several logistical challenges. Some of the email addresses provided by the colleges were inaccurate, while others belonged to students who no longer actively used those accounts. Additionally, a portion of the survey invitations may have been filtered into recipients’ spam folders, further limiting visibility and participation.
- The 6.3% of the participants who were under the influence of alcohol was self-reported.

Discussion – line 129 -lower limbs were most affected, but in line 190 -Injury patterns showed the upper limb was most affected (49.9%), is this from different study or the narration is inconsistent.

- I reviewed the section in question. The statement in line 129, indicating that the lower limb was most commonly affected, reflects the findings of my own study. In line 190, the reference to two studies from Nepal, one from Nigeria, and one from Laos also supports this pattern, showing consistent results across different settings. The discrepancy was due to a typing error, which I’ve now corrected.

Recommendations- are general – what u can say (my suggestions only) is drive for better licensing program, proper multi-level data capture for RTA and RTI.
Most important
What are the infrastructure data you have collected for RTA? road banking, road marking etc.
Environmental factors – rain/fog/road repair / road illumination at a particular place,
You can use some CI for proportions presented.

- Thank you for your thoughtful feedback. The recommendation for better licensing program has been included in the manuscript. The environmental factors and driving behaviors were indeed explored as part of the original thesis, but they are being developed into a separate manuscript due to space constraints in the current paper. This study was already quite extensive, so we’ve planned a follow-up publication that will specifically address infrastructure, environmental influences, and behavioral aspects in more detail. I also appreciate your suggestion regarding recommendations and will incorporate more targeted and actionable points as advised.

Some good data is with this team needs proper presentation.

Reviewer #2: REVIEW

Motorization and Its Consequences: A Mixed-Methods Study on the Epidemiology, Impact, and Stakeholder Perspectives of Road Traffic Injuries Among Undergraduate Students in Nepal

Over all: This study is relevant as it addresses the rising burden of road traffic injuries among youth in Nepal, a vulnerable yet often overlooked population in public health policy. With the suggested changes, it can be considered for publication.

Abstract
Line 29: Methodology: to check this statement ’Methodology: The quantitative section was conducted among undergraduate students in the four colleges in Kathmandu district.’ Quantitative section?

- The methodology section has been corrected from section to data collection

Introduction:
Line 52 and 54: ‘Injuries are disproportionately high
among males, disadvantaged groups,’ add why they are disadvantaged groups.

- This line is cited from WHO *Powered Two- and Three-Wheeler Safety Manual* (2022), and it refers to populations who face systemic barriers to safety and healthcare access, and are disproportionately affected by road traffic injuries due to socioeconomic, geographic, or structural vulnerabilities, limited access to safety infrastructure, protective gear, legal protections, emergency services, Such as informal workers, delivery riders, low income population and rural or peri urban residents, which has been simplified and added to the manuscript for clarity.

Line 77, 78: It is mentioned 28 classes, specify what classes are. ‘Participation was voluntary and gave informed consent ‘check the grammar of this statement

- Line 78 the line has been corrected
- Line 77: the details of the 28 classes had been added:
- The study included a total of 28 classes across four colleges—Kathmandu Medical College and Nepal medical college (Medicine ( 1^st^, 2^nd^, 3^rd^ 4^th^ year, Dental (, 2^nd^, 3^rd^ 4^th)^, BSc Nursing , 2^nd^, 3^rd^), College (Developmental Studies, (, 2^nd^, 3^rd^ 4^th)^ Finance ( , 2^nd^, 3^rd^ 4^th^), and St. Xavier’s College (Bachelor in Social Work, , 2^nd^, 3^rd^ 4^th^)—with students enrolled from first to fourth year in each stream

Methodology: Add timeline/ duration of the study

- The data collection was done from December 2020 to February 2021, and it has been added in the manuscript

Results: Line 168, Check the grammar. Table 23 appears before table 4. Where are table 2 and 3?

- The errors in the number of the tables have been corrected. Table 23 is actually table 2 and table 4 became table 3.

Discussion:
Line 198 and 199: ‘no statistically significant association
was found between licensing status and RTI occurrence’. This is not reflected in results.

- The aspects of driving behavior and licensing will be addressed in a separate manuscript derived from the same thesis. Therefore, the paragraph discussing license usage will be removed from the current paper to avoid overlapping.

‘The themes and recommendations from the qualitative study’ should be better discussed in this section. The discussion could be strengthened further through more robust comparisons with national and international studies, which would help contextualize the findings, highlight unique challenges in the Nepali context, and support evidence-based recommendations.

- The discussion has been changed, it now includes comparison with national and international studies and reports, and the discussion now holds themes and recommendations from both quantitative and qualitative data.
