# Supplementary material for: Motorization and its consequences: A mixed-methods study on the epidemiology, impact, and stakeholder perspectives of road traffic injuries among undergraduate two-wheeler riding students in Nepal
Source: PLoS One. 2026 May 27;21(5):e0341275. doi: 10.1371/journal.pone.0341275 (PMC13215494; doi:10.1371/journal.pone.0341275)
Supplement: Attachment — Submitted filename: Response to Reviewers.docx [file pone.0341275.s005.docx]

Please find all the point by point response to the reviewer’s comments below:

- Reviewer #2: REVIEW
- Abstract : Line 29: Methodology: to check this statement ’Methodology: The quantitative section was conducted among undergraduate students in the four colleges in Kathmandu district.’ Quantitative section?

The line has been corrected to qualitative study

- Introduction: Line 52 and 54: ‘Injuries are disproportionately high
  among males, disadvantaged groups,’ add why they are disadvantaged groups.

This line is cited from WHO *Powered Two- and Three-Wheeler Safety Manual* (2022), and it refers to populations who face systemic barriers to safety and healthcare access, and are disproportionately affected by road traffic injuries due to socioeconomic, geographic, or structural vulnerabilities, limited access to safety infrastructure, protective gear, legal protections, emergency services, Such as informal workers, delivery riders, low income population and rural or peri urban residents, which has been simplied and added to the manuscript for clarity with reference.

- Line 77, 78: It is mentioned 28 classes, Specify what are classes. ‘ Participation was voluntary and gave informed consent ‘ check the grammar of this statement

Line 78 the line has been corrected

- Line 77 : the details of the 28 classes had been added :

The study included a total of 28 classes across four colleges—Kathmandu Medical College and Nepal medical college (Medicine ( 1^st^, 2^nd^, 3^rd^ 4^th^ year, Dental (, 2^nd^, 3^rd^ 4^th)^, BSc Nursing , 2^nd^, 3^rd^), College (Developmental Studies, (, 2^nd^, 3^rd^ 4^th)^ Finance ( , 2^nd^, 3^rd^ 4^th^), and St. Xavier’s College (Bachelor in Social Work, , 2^nd^, 3^rd^ 4^th^)—with students enrolled from first to fourth year in each stream

- Methodology: Add timeline/ duration of the study

The data collection was done from December 2020 to February 2021.

- **Results: Line 168, Check the grammar. Table 23 appears before table 4. Where are table 2 and 3?**
  The errors in the numbering of the tables has been corrected, The table 23 is actually table 2 and table 4 has now been corrected labelled as table 3.
- Discussion:
  Line 198 and 199: ‘no statistically significant association
  was found between licensing status and RTI occurrence’. This is not reflected in results.

The aspects of driving behavior and licensing are being addressed in a separate manuscript derived from the same thesis. Therefore, the paragraph discussing license usage will be removed from the current paper as it has not been mentioned in the manuscript earlier.

Although licensing was a part of the thesis, it has not been mentioned earlier in the current manuscript. Therefore, to maintain consistency and avoid introducing unsupported findings, the paragraph discussing license usage has been removed.

- ‘The themes and recommendations from the qualitative study’ should be better discussed in this section. The discussion could be strengthened further through more robust comparisons with national and international studies, which would help contextualize the findings, highlight unique challenges in the Nepali context, and support evidence-based recommendations.

The discussion has been fully changed, it now includes comparison with national and international studies and reports and the discussion now holds themes and recommendations from both quantitative and qualitative data.

Please find all the point by point response to the reviewer’s comments below:

Reviewer #3:

- The presentation has improved a lot at the same time - follow-up publication can not be justification for clarity of this manuscript .

-Thank you for your thoughtful feedback. In this study, we primarily focused on the epidemiology and impact of road traffic injuries among undergraduate two‑wheeler riders. While infrastructure, environmental, and behavioral factors such as road condition, traffic density, time and location of incidents, atmospheric conditions, mechanical aspects of two‑wheelers, and driving behaviors (speeding, alcohol use, passenger distraction, helmet use, fatigue, extended riding duration, riding while unwell, carrying heavy loads, and traffic law obedience) were explored during the original thesis work, they have not been included in this manuscript due to space constraints. The current paper was already extensive, and we prioritized clarity by limiting the scope to the most relevant findings.

- further the conclusion and recommendation are general but not restricted to your findings - you can also suggest for better clinical facility to reduce morbidity and disability among affected .

-The Conclusion and the recommendations have been changed to go beyond the findings of this paper and incorporated recommendations related to better facilities to reduce disability.

Reviewer #3:

Abstract: 5 key recommendations that came forward; change it it 5 key recommendations that emerged

-This has been addressed in the manuscript

Line 79: Qualitative study was conducted; is it qualitative or quantitative, because rest of the paragraph describes the quantitative data collection process.

-The error has been corrected was describing the quantitative data collection process.

Line 82-84: very colloquial.

-This has been addressed in the manuscript

Line 85-86: The data 85 was collected from students enrolled in first to fourth year across 28 academic classes across four colleges, Kathmandu Medical College, Nepal Medical College, College, reword to remove the word appearing across twice.

-This has been addressed in the manuscript

Line 95-99: It appears that the qualitative and quantitative data were collected from different populations. Additionally, the qualitative component involved only five participants; however, the process by which data saturation was determined is not clearly described. Although the study is presented as a mixed-methods design, the Methods section lacks clarity regarding the specific mixed-methods design employed, the sampling strategies used for each component & in the perspective of mixed methods research, and how the qualitative and quantitative strands were integrated.

Thank you for this observation. The study employed an explanatory sequential mixed‑methods design, which is a two‑phase approach. In the first phase, quantitative data were collected from undergraduate students to determine the prevalence of road traffic injuries and associated risk factors. In the second phase, qualitative data were gathered through five key informant interviews with experienced stakeholders (e.g., college coordinator, emergency physician, traffic police officer, former Director‑General of Transport Management, and judge). These interviews were not intended to achieve thematic saturation in the traditional sense, but rather to capture expert perspectives that could contextualize and extend the quantitative findings.

The sampling strategies therefore differed by strand: the quantitative component used student participants to establish prevalence and risk factors, while the qualitative component purposively selected experts to provide recommendations and system‑level insights. Integration occurred through the explanatory sequential design, where qualitative findings were explicitly used to interpret and build upon the quantitative results, thereby strengthening the overall interpretation and providing actionable guidance.

**Data Analysis:**

line 101-107: The data analysis software used both for qualitative and quantitative data analysis to be specified.

-This has been addressed in the manuscript

**Results:**
Line 112: was 1878, this number is 1879 in the methods section, (line number 89), be consistent with this data.

-This has been addressed in the manuscript, the number is 1879. The consistency has been checked throughout the manuscript.

Line 114: and 65.6.6 (what is this two decimal number to be clarified)

-It has been corrected, it was a typing error

What does table 1 describe, the table title should be appropriate, if it is of respondents then the n=1217, if it is the one using two wheeler, the findings reveal 578 (line 113), but the table has n=579, to provide titles for all the tables.

-This has been addressed in the manuscript, the number is 579. The consistency has been checked throughout the manuscript.

Line 154: The expansion of abbreviation NPR is not found in the prior to the usage

-It was been expanded.

Table 3: The themes identified in the qualitative analysis need to be described in greater detail to enhance better understanding. Themes and related verbatim can be jotted together. However, the emerged recommendation require additional description and details with the base for this.
Qualitative data/verbatims clearly lack saturation.

-I would like to clarify that the qualitative component of this study was explicitly designed to elicit recommendations for reducing road traffic injuries from key stakeholders. The recommendations that emerged were subsequently organized into five thematic areas : education and awareness, reducing dependency on two‑wheelers, strengthening the role of traffic police, legal reforms, and licensing systems. Within each theme, the recommendations are supported by verbatim quotes from informants to illustrate the rationale and provide context for the suggestions. We acknowledge that the qualitative sample was limited to five informants, and therefore the verbatims may not demonstrate full saturation. However, the intent of this phase was not to achieve thematic saturation in the traditional sense, but rather to capture expert perspectives that complement the quantitative findings. The integration of these two strands follows the explanatory sequential mixed‑methods design, where the qualitative insights build directly upon the quantitative results to provide depth and actionable guidance.

For clarity and uniformity, the Results section should report categorical variables using the n (%) format.
The Results section shows inconsistency in the tense used for reporting findings and requires revision to ensure uniform tense throughout

-This has been revised thoroughly and has been uniform in reporting categorical variable has been done in n(%) format.

**Discussion:**
line 184: This study applied the Safe Systems Approach, and the this approach was not introduced in the background or methodology, and all these need to be consistently aligned throughout.

-I agree with you, the safe system approach was not consistently aligned so it has reworked upon.

RTIs/RTI need not be expanded in each section : it has been corrected
References: 36, is not found cited
-It has now been cited

Dear Academic Editor and Reviewers,

We sincerely thank you for your thoughtful and constructive feedback on our manuscript. “Motorization and Its Consequences: A Mixed-Methods Study on the Epidemiology, Impact, and Stakeholder Perspectives of Road Traffic Injuries Among Undergraduate Two-Wheeler Riding Students in Nepal”

We have carefully considered each of your comments and revised the manuscript accordingly. Below, we provide a point-by-point response to the editorial requirements and reviewer comments. All changes have been incorporated into the revised manuscript, and we believe these revisions have significantly improved the clarity and quality of our work.

We are grateful for the opportunity to revise our manuscript and for the valuable feedback provided. We hope the revisions meet your expectations and look forward to your favorable consideration.

Sincerely,

Dr. Nikita Bhattarai

(on behalf of all autho
